# Supplementary material for: Rapid turnover of life-cycle-related genes in the brown algae
Source: Genome Biol. 2019 Feb 14;20:35. doi: 10.1186/s13059-019-1630-6 (PMC6374913; doi:10.1186/s13059-019-1630-6)
Supplement: Supplementary file 3 — Protein alignments, corresponding Gblocks html file and CDS sequences for the lineage conserved gametophyte-biased genes. (ZIP 2109 kb) [file 13059_2019_1630_MOESM3_ESM.zip › Con_GA_alignments/Ec-26_000580.aa_ali.fasta-gb.htm]

Ec-26\_000580.aa.fasta.trans.aa\_ali.fasta


## Gblocks 0.91b Results

Processed file: **Ec-26\_000580.aa.fasta.trans.aa\_ali.fasta**  
Number of sequences: **2**  
Alignment assumed to be: **Protein**  
New number of positions: **790** (selected positions are underlined in blue)

```
                         10        20        30        40        50        60
                 =========+=========+=========+=========+=========+=========+
Ec-26_000580     MLSGTRTSIESPWRFVLGCLSTRTGSVDPAPRVKMLDTVLLDGTGGGQAEIFFFTAADGT
MSTRG.32743.1    ------------------------------------------------------------
                                                                             


                         70        80        90       100       110       120
                 =========+=========+=========+=========+=========+=========+
Ec-26_000580     VRRKGERFLSLKAIEKELRSRATTFNKNAGHGSGPGDSTLQTAEQVIAMARRKTGEYFPI
MSTRG.32743.1    ------------------------------------------------------------
                                                                             


                        130       140       150       160       170       180
                 =========+=========+=========+=========+=========+=========+
Ec-26_000580     TSKMLHTIVKGGVVPADFDCIYPFKGAKDFDITAEFANYQHQYILDDTGGGVSTGTTHRR
MSTRG.32743.1    ------------------------------------------------------------
                                                                             


                        190       200       210       220       230       240
                 =========+=========+=========+=========+=========+=========+
Ec-26_000580     VALETSGSKQGQGAGAGAQRQNLLLSKNARINTEVVATVRSVVIAIERSKLCRVLRLDTE
MSTRG.32743.1    ------------------------------------------------------------
                                                                             


                        250       260       270       280       290       300
                 =========+=========+=========+=========+=========+=========+
Ec-26_000580     FVLDGNDELWLVGVTCCTVAARSTISGAQQLGDNGPQGASFLKEEERTLATELRHRKHEA
MSTRG.32743.1    ------------------------------------------------------------
                                                                             


                        310       320       330       340       350       360
                 =========+=========+=========+=========+=========+=========+
Ec-26_000580     DQISGVLGDEKFSQLLRSVGYHFPEKSRAVARRRRHRPRPIVDSTSRSSTIPRNDNDEYR
MSTRG.32743.1    ------------------------------------------------------------
                                                                             


                        370       380       390       400       410       420
                 =========+=========+=========+=========+=========+=========+
Ec-26_000580     ARETDGDSVALSQRVLPNTGTRSQRTGSREAVHAGMASSDWADPGPGEGSATSDFGDKDG
MSTRG.32743.1    ------------------------------------------------------------
                                                                             


                        430       440       450       460       470       480
                 =========+=========+=========+=========+=========+=========+
Ec-26_000580     GSGAASRSPSPPRSTEHFDSSVAKLDQAATNRIYGSTQLEGCRGDFCDFDVGKRVREEEN
MSTRG.32743.1    ------------------------------------------------------------
                                                                             


                        490       500       510       520       530       540
                 =========+=========+=========+=========+=========+=========+
Ec-26_000580     HRRFAEKAARAAGAPNGLVESLSTLRRKILSMESGDLGDGPSASATPAEVREGGRGPRVA
MSTRG.32743.1    ------------------------------------------------------------
                                                                             


                        550       560       570       580       590       600
                 =========+=========+=========+=========+=========+=========+
Ec-26_000580     RHTDATSERLDIPYKAVVQARQERPLVEAFLRRYARGEDGAYHRYLDGGQGDEPYLVGGK
MSTRG.32743.1    ------------------------------------------------------------
                                                                             


                        610       620       630       640       650       660
                 =========+=========+=========+=========+=========+=========+
Ec-26_000580     YPGVYYRPVKVCLNCHMVYTLLDEARARALRTASRTCCAGGGDRRHPPTPRLTLGQPSCD
MSTRG.32743.1    ------------------------------------------------------------
                                                                             


                        670       680       690       700       710       720
                 =========+=========+=========+=========+=========+=========+
Ec-26_000580     GKGAGSVPTAKMLLTRAGENDNKDGRGHKESPIAAEESVPAVSASSEGHEHSSLAVSLSE
MSTRG.32743.1    ------------------------------------------------------------
                                                                             


                        730       740       750       760       770       780
                 =========+=========+=========+=========+=========+=========+
Ec-26_000580     ARRAMDVISQGDISELRSLARPPSAVVHVVSIALLLLEGKTTEKAAAAPVSWAIARAAMC
MSTRG.32743.1    ------------------------------------------------------------
                                                                             


                        790       800       810       820       830       840
                 =========+=========+=========+=========+=========+=========+
Ec-26_000580     RADLLPRLRALDPRAVAPQQLSSLGPALERSSLDPAVVRPLCNAAGNLCLWVLGVFQANR
MSTRG.32743.1    ------------------------------------------------------------
                                                                             


                        850       860       870       880       890       900
                 =========+=========+=========+=========+=========+=========+
Ec-26_000580     WLTGSGHSRTNVVPVDGDIRRWGYDHVRKHRGIAVVQRQQPFPKQKSPRRTRWASSSRAP
MSTRG.32743.1    -------------------------------------------------RARWGSPQGTK
                                                                  ###########


                        910       920       930       940       950       960
                 =========+=========+=========+=========+=========+=========+
Ec-26_000580     AGRRRCRMENHAVRAVPSNNGRGGREAGSSVSPERTSTVGFGAFGPATASPELGGGANSG
MSTRG.32743.1    TQRKRDRTESHTERHAPTKSNKYGRLEGGSVSLERATVRGFGEFGPATASPTLAGGNDAG
                 ############################################################


                        970       980       990      1000      1010      1020
                 =========+=========+=========+=========+=========+=========+
Ec-26_000580     FGVSSDRDDAVGATSG--APASSSDKRSPCRRKKNLGDRVAAQAFASGRLANAGQCDAPE
MSTRG.32743.1    FRVSAGNDSGTDNDSAPTPAASLSSDRSPHRRRKKVGGRVAAQAFASDRLANGGQSALPE
                 ###############     ########################################


                       1030      1040      1050      1060      1070      1080
                 =========+=========+=========+=========+=========+=========+
Ec-26_000580     ASSGKHFVCSDGRTRLPYRVCGNPGTSSGIAESCNFVVVHDFFDNVDKTEVLFRPVTRKH
MSTRG.32743.1    VASLETFLCSDGRSRLPYRVCGNPGASSGVADSCNFVVVHDFFDNLDKTEVFFRPITRRH
                 ############################################################


                       1090      1100      1110      1120      1130      1140
                 =========+=========+=========+=========+=========+=========+
Ec-26_000580     RGCRVLAFSYPGQAGTVFRVPPSMVALASPEGNATRSNGGGAHGLGSSHSGVGSGGGGEK
MSTRG.32743.1    RGCRVLTFSYPGQAGTVFRVPQSLANLSP---SGSKEEPVCLRSGGGNRAGAGRGDDADV
                 ###########################                  ##########     


                       1150      1160      1170      1180      1190      1200
                 =========+=========+=========+=========+=========+=========+
Ec-26_000580     VRKEVPNNAFLAPRLHELLQHVHSVGEMSLTAPFHLVGIGNGMATASAFALRYGDHPLYR
MSTRG.32743.1    IQNDVPNNTFIAPRLHELLQHVHSVGQMSLAVPFHLVGIGNGMATAAAFALSYGDHPLYR
                     ########################################################


                       1210      1220      1230      1240      1250      1260
                 =========+=========+=========+=========+=========+=========+
Ec-26_000580     SSIRSVVSINGFSSVDSQLAAILHSSLNAFATLPPARPDLPVSFMSRYIFSDDYLRKVGR
MSTRG.32743.1    SSLRSVVSINGFSSVDSQLAAILHSSLNAFATLPSTRPDLPVSFMSRYMFSEDYLKKVGR
                 ############################################################


                       1270      1280      1290      1300      1310      1320
                 =========+=========+=========+=========+=========+=========+
Ec-26_000580     DLALGIYTAVANPVSLEGRHLLCNSALLHEDMSAEVGALGVPIVLLQSTEDMLVNPANVD
MSTRG.32743.1    NLALGISTAVANPISLEGRHLLCSSALLHEDLSAEVGSLGVPIVLLQSTEDVLVNPANVD
                 ############################################################


                       1330      1340      1350      1360      1370      1380
                 =========+=========+=========+=========+=========+=========+
Ec-26_000580     PFLRGRSSTHHFWSHEFRDGRSGAGSDSELTSSAAAAEAARGSSVYGRKGLTDLLRAMSR
MSTRG.32743.1    PFLRGRSSTHHFWSHEFRDGGGVVGMGGDLDASSIAPVS--QSSVYGRKGLTDLLRALSK
                 ####################################      ##################


                       1390      1400      1410      1420      1430      1440
                 =========+=========+=========+=========+=========+=========+
Ec-26_000580     PRGTFVAWVRAGHEVCQEGKRAVIDLLDVLAKPTPAYTGVDEVDVLQGEAEGAATLGLYP
MSTRG.32743.1    PRGTFVAWVRAGHEVCQEAKRAVIDLLDVLAKPTLRHTGVSEADVLRGNPTGATTVGLYP
                 ############################################################


                       1450      1460      1470      1480      1490      1500
                 =========+=========+=========+=========+=========+=========+
Ec-26_000580     SGEWVARVNKRGGGPANAAEVATSRTG-----------DFDDSEGDDAAMSADRRRREEK
MSTRG.32743.1    SGEWVARVNRRGGRQGEKSEQLPPRRGTGADACVAAAVDMKNDGGSEESSADTPVSVDHG
                 ###########################                                 


                       1510      1520      1530      1540      1550      1560
                 =========+=========+=========+=========+=========+=========+
Ec-26_000580     ERLDCDG-IGEEVAEQEGSG--LQAAASPFPRDLSIPTLPASVALGHRTPNTSPIKRSHA
MSTRG.32743.1    DREKSHKQRGEDLSDRETRDRVGPPATAPFPRDITIPALPASIALAHRGLHTSPIKRSNV
                                          ###################################


                       1570      1580      1590      1600      1610      1620
                 =========+=========+=========+=========+=========+=========+
Ec-26_000580     ATSGAVNAAGRGRGAHHTVGGRSGSSRRQARSVDAFGVGDDKDVEPHEGRQARGRGRGRD
MSTRG.32743.1    VTSSSLADGSAAKGARHTFEGMRGS-RNQSRRVDAFGLTNDNDTERGRRRAAPGHKRPRG
                 ###          ############ ##################################


                       1630      1640      1650      1660      1670      1680
                 =========+=========+=========+=========+=========+=========+
Ec-26_000580     RRALDARERDSGAVGSPLAAEH-GYRRRPKVVWKDNTPQEAVEQVAPTTAPFLTRRAHGD
MSTRG.32743.1    RGSSDVVRRDVGAANGTLLAESDRHRRVPKVVWKDSTPLANVGGEE-------RAHNEGD
                 #####################    #################                  


                       1690      1700      1710      1720      1730      1740
                 =========+=========+=========+=========+=========+=========+
Ec-26_000580     EDEKGG---AGEKGYSASYFPTAAVLYDG----EPTN----------RPTALRD------
MSTRG.32743.1    RDERKGEGTIRTKDYSTSYFPEAALVYDGGSSLSPTSGSREGGSCHHPPIPLSDESANAL
                             #################                               


                       1750      1760      1770      1780      1790      1800
                 =========+=========+=========+=========+=========+=========+
Ec-26_000580     -----REDPWDLLSNPPSLEFPLSGEHQRGNRRWVVNKPP-------AGENGGQGEVSPT
MSTRG.32743.1    LPSSGYGDTWDLINDPPSLELPASGELQRGNRRWLIEKKSDSTATAGRGESGGSEGAPDS
                        ###############################          ############


                       1810      1820      1830      1840      1850      1860
                 =========+=========+=========+=========+=========+=========+
Ec-26_000580     SPTSSPTAAATAGATSSIGHGSD-----SPPLADLLEAEASLEGRLCEARRRAAERLVRE
MSTRG.32743.1    SANTTATAAATAGSTRTNNTGADGAGSATLPLRDLLEAEASLESRLCEARRMAAERLARE
                 #######################       ##############################


                       1870      1880      1890      1900      1910      1920
                 =========+=========+=========+=========+=========+=========+
Ec-26_000580     EADAERLIAGITREQQARGREFAEEDRQMIADLEAQLAAERRARAPADLQRAVDGVDVDD
MSTRG.32743.1    EAEAERRIAGITEEQX--------------------------------------------
                 ###############                                             


                       1930      1940      1950      1960      1970      1980
                 =========+=========+=========+=========+=========+=========+
Ec-26_000580     AIVRGGLVASKSPSSPPTSSASTRGRKDNCDGVAEGEAVGGVGGAFDASSTSPPSSFPVS
MSTRG.32743.1    ------------------------------------------------------------
                                                                             


                       1990      2000      2010      2020      2030      2040
                 =========+=========+=========+=========+=========+=========+
Ec-26_000580     AMPPLDYSPLDDLPEQLQRATDAYSLMDDAARDEAEMLRIRKATGGGAMSVEEFQRDQAA
MSTRG.32743.1    ------------------------------------------------------------
                                                                             


                       2050      2060      2070      2080      2090      2100
                 =========+=========+=========+=========+=========+=========+
Ec-26_000580     AAAEAAAWRLGTKKAFRKRSKSELERARVEAALRFQPLVRGVLGRKRARQLRLQRDEERQ
MSTRG.32743.1    ------------------------------------------------------------
                                                                             


                       2110      2120      2130      2140      2150      2160
                 =========+=========+=========+=========+=========+=========+
Ec-26_000580     LSAAATKVQAVARGHLAKEHARAAREAAVAELVLGGSAIRLQSAGRGMLGRRRAARRRRQ
MSTRG.32743.1    ------------------------------------------------------------
                                                                             


                       2170      2180      2190      2200      2210      2220
                 =========+=========+=========+=========+=========+=========+
Ec-26_000580     VMAGAIQRCYRGHLGRRSAANQRALLETLRRRNLATTRIQAWWRCKLAMDGYASGRATSI
MSTRG.32743.1    ------------------------------------------------------------
                                                                             


                       2230      2240      2250      2260      2270      2280
                 =========+=========+=========+=========+=========+=========+
Ec-26_000580     AAIEIQRCYRGMIGRKKASRRLEWEKSEPGPERLKLGVRLIEDSKNAFEAQRMEIAALHT
MSTRG.32743.1    ------------------------------------------------------------
                                                                             


                       2290      2300      2310      2320      2330      2340
                 =========+=========+=========+=========+=========+=========+
Ec-26_000580     AEERAAIRTSRIRKELGASEKELTALEREMHEIDHIEGQLSQLNHQRNMVQLGLMQAGET
MSTRG.32743.1    ------------------------------------------------------------
                                                                             


                       2350      2360      2370      2380      2390      2400
                 =========+=========+=========+=========+=========+=========+
Ec-26_000580     MPGIDTPALSGGGAGGSGGVGGGVRSGDNVRDAADKNLGFAIEMQIQVKRAEREKKRQEL
MSTRG.32743.1    ------------------------------------------------------------
                                                                             


                       2410      2420      2430      2440      2450      2460
                 =========+=========+=========+=========+=========+=========+
Ec-26_000580     EVDFRGVRQEVDLKRRELDRVSAAITEIENTRERKTVEFRRMQANLMELLREQKLELDAV
MSTRG.32743.1    ------------------------------------------------------------
                                                                             


                       2470      2480      2490      2500      2510      2520
                 =========+=========+=========+=========+=========+=========+
Ec-26_000580     KEKGIQLEVATATSAAAASATAQRARDHEERSSEMYSQTEELMKFQFMSMSLSYFSSLNM
MSTRG.32743.1    ------------------------------------------------------------
                                                                             


                       2530      2540      2550      2560      2570      2580
                 =========+=========+=========+=========+=========+=========+
Ec-26_000580     LKTMRDINADTTTAAVTSSADAAAAAAASAAAANIPAIKAGKIMESVADVTTQEIGRKNK
MSTRG.32743.1    ------------------------------------------------------------
                                                                             


                       2590      2600      2610      2620      2630      2640
                 =========+=========+=========+=========+=========+=========+
Ec-26_000580     VLQEKMEAQEEMEEANAHPFPPEVRFWTKEDVGFFLTTLGLRQYRAAFEEAAVDGDFLLA
MSTRG.32743.1    ------------------------------------------------------------
                                                                             


                       2650      2660      2670      2680      2690      2700
                 =========+=========+=========+=========+=========+=========+
Ec-26_000580     LDANDCADVLGVEHALHSKKLFLAIDKLRPLGAEERRKKAVVEREEFADQNRGDTAPDVE
MSTRG.32743.1    ------------------------------------------------------------
                                                                             


                       2710      2720      2730      2740      2750      2760
                 =========+=========+=========+=========+=========+=========+
Ec-26_000580     TVFSQVRNGRLKRVEDSLEKGFSIDTTDEHGNTALLVACQNCNVQLCEMLLRRGADVNHR
MSTRG.32743.1    ------------------------------------------------------------
                                                                             


                       2770      2780      2790      2800      2810
                 =========+=========+=========+=========+=========+==
Ec-26_000580     NGRGNTPLHYAMAYDTQGVLGEMLISRGGDDTVTNRDGLSCYDGLGGTPPSA
MSTRG.32743.1    ----------------------------------------------------
```

```
Parameters used
Minimum Number Of Sequences For A Conserved Position: 2
Minimum Number Of Sequences For A Flanking Position: 2
Maximum Number Of Contiguous Nonconserved Positions: 8
Minimum Length Of A Block: 10
Allowed Gap Positions: None
Use Similarity Matrices: Yes
```

```
Flank positions of the 13 selected block(s)
Flanks: [890  975]  [981  1107]  [1126  1135]  [1145  1356]  [1363  1467]  [1526  1563]  [1574  1585]  [1587  1641]  [1646  1662]  [1693  1709]  [1748  1778]  [1789  1823]  [1831  1875]  

New number of positions in Ec-26_000580.aa.fasta.trans.aa_ali.fasta-gb:  790  (28% of the original 2812 positions)
```
